# Perinatal Asphyxia May Influence the Level of Beta-Amyloid (1-42) in Cerebrospinal Fluid: An Experimental Study on Newborn Pigs

**DOI:** 10.1371/journal.pone.0140966

**Published:** 2015-10-26

**Authors:** Torkil Benterud, Leonid Pankratov, Rønnaug Solberg, Nils Bolstad, Anders Skinningsrud, Lars Baumbusch, Leiv Sandvik, Ola Didrik Saugstad

**Affiliations:** 1 Dept of Pediatric Research, Oslo University Hospital, Oslo, Norway; 2 Dept of Medical Biochemistry, Oslo University Hospital, Oslo, Norway; 3 Dept Multidisciplinary Lab Med & Med Biochem, Akershus Univ Hosp, Lørenskog, Norway; 4 Dept of Biostatistics and Epidemiology, Oslo University Hospital, Oslo, Norway; NIH, UNITED STATES

## Abstract

**Objective:**

Total tau (T-tau), phosphorylated tau (p-Tau) and Beta-Amyloid 1–42 (AB42) in Cerebrospinal Fluid (CSF) are useful biomarkers in neurodegenerative diseases. The aim of the study was to investigate the role of these and other CSF biomarkers (T-tau, p-Tau, AB42, S100B and NSE), during hypoxia-reoxygenation in a newborn pig model.

**Design:**

Thirty newborn pigs were included in a study of moderate or severe hypoxia. The moderate hypoxia group (n = 12) was exposed to global hypoxia (8% O_2_) until Base excess (BE) reached -15 mmol/l. The pigs in the group exposed to severe hypoxia (n = 12) received 8% O_2_ until BE reached -20 mmol/l or mean Blood Pressure fell below 20 mm Hg, The control group (n = 6) was kept at room air. For all treatments, the CSF was collected at 9.5 hours after the intervention.

**Results:**

The level of AB42 in CSF was significantly lower in the pigs exposed to severe hypoxia compared with the control group, 922(SD +/-445)pg/ml versus. 1290(SD +/-143) pg/ml (p<0.05), respectively. Further, a non-significant reduction of AB42 was observed in the group exposed to moderate hypoxia T-tau and p-Tau revealed no significant differences between the intervention groups and the control group, however a significantly higher level of S100B was seen in the CSF of pigs receiving hypoxia in comparison to the level in the control group. Further on, there was a moderate negative correlation between the levels of AB42 and S100B in CSF, as well as a moderate negative correlation between Lactate in blood at end of hypoxia and AB42 in CSF.

**Interpretation:**

This is the first study to our knowledge that demonstrated a significant drop in AB42 in CSF after neonatal hypoxia. Whether or not this has an etiological basis for adult neurodegenerative disorders needs to be studied with additional experiments and epidemiological studies. AB42 and S100B are significantly changed in neonatal pigs subjected to hypoxia compared to controls and thus may be valuable biomarkers of perinatal asphyxia.

## Introduction

Intrapartum events are among the most common causes of neonatal death with more than 800,000 annual cases worldwide [1].

Even though the majority of the children exposed to severe perinatal asphyxia will survive, many of them will suffer from long-term sequelae, such as cerebral palsy and cognitive deficits.

In severe cases, perinatal asphyxia may lead to Hypoxic-Ischemic Encephalopathy (HIE), which may cause permanent neurological damage.

Proteins, such as S100B and Neuron specific Enolase (NSE), released into the cerebrospinal fluid (CSF) during neuronal injury, might be useful as biomarkers in reflecting disease severity and predicting the clinical outcome after perinatal asphyxia [2].

We addressed the question whether total-tau (T-tau), phospho-Tau (p-Tau) and Beta-Amyloid 1–42 (AB42), biomarkers of adult neurodegenerative disorders, could serve as markers of perinatal asphyxia [3,4,5].

In pediatric populations altered T-tau levels in CSF have been found in patients with brain tumors [6] and West syndrome (Infantile spasms) [7]. Magnoni et al.(2012) found that T-tau in the brain extracellular space was increased and negatively correlated with Beta-Amyloid levels in the extracellular space after traumatic brain injury and that T-tau may be helpful when predicting the clinical outcome [8].

In two retrospective studies Rondell et al. and Zetterberg et al. showed increased serum levels of Tau-protein and AB42, respectively, after hypoxia due to Cardiac arrest [9, 10]

Few, if any, experiments have been conducted to investigate if there is an association between asphyxia in the neonates and the levels of these markers.

In addition to T-tau, p-Tau and AB42, we also addressed the question of how NSE and S100B were affected in our model of neonatal hypoxia-reoxygenation.

## Objective

The objective was to determine a possible correlation between the levels of CSF T-tau, p-Tau, AB42, S100B and NSE after hypoxia-reoxygenation in the newborn pig and establish a possible association between perinatal hypoxia-reoxygenation and any of these markers.

## Materials and Methods

### Study design

Thirty newborn pigs, age12–36 hours, Hb > 5g/dl and in good general condition were included in the study.

The pigs were given fentanyl 25microg/kg, midazolam 1.0mg/kg and pentobarbitone 20mg/kg intravenously as bolus injections for induction of anaesthesia before they were intubated and placed on their backs and washed for sterile procedures. Anaesthesia was maintained by a continuous infusion of fentanyl (50 microg/kg/h) and midazolam (0.25 mg/kg/h; IVAC P2000 infusion pump). When necessary, a bolus of fentanyl (10 microg/kg) and midazolam (1 mg/kg) were administered (need for medication being defined as shivering, increase in blood pressure and/or pulse and increased tone assessed by passive movements of the limbs). Pentobarbitone (2.5 mg/kg) was a few times added if there was increased muscular tone that did not respond to fentanyl or midazolam. A continuous iv. infusion, Salidex: saline 0.3% and glucose 3.5%, 10 ml/kg/h was given until start of hypoxia. From 15 min after end of hypoxia the infusion was continued at 5 ml/kg/h.

The pigs were ventilated with a pressure-controlled ventilator (Babylog 8000+; Drägerwerk, Lübeck, Germany) IMV mode, humidification by Fisher and Paykel MR730, 39°C. Normoventilation (arterial carbon dioxide tension (PaCO_2_) 4.5–6.0 kPa) was achieved by adjusting the peak inspiratory pressure or ventilatory rate.

### Surgical preparation

The left jugular vein was cannulated with an arterial canula with FloSwitch (20G/1,10mm x 45mm. B.D. Faraday Road, Swindon, UK), and the right carotic artery was cannulated using a venflon (BD Venflon Pro, 22GA, 0,9mm x 25mm. Becton Dickinson Infusion Therapy AB, Helsingborg, Sweden). Both procedures were conducted under sterile conditions, and the cannulas were sutured to the skin. The animals were thereafter placed in a prone position for the rest of the experiment. Rectal temperature was maintained between 38.5 and 39.5°C with a heating blanket and a radiant heating lamp. Mean arterial blood pressure (MABP) was measured continuously in the right carotic artery using BioPac systems MP150-CE.

### Experimental protocol

Twelve pigs were included in each experimental group and six pigs were in the control group. After 1 hour of stabilization the pigs in the intervention groups went through global hypoxia and reoxygenation with air.

**Fig 1:**
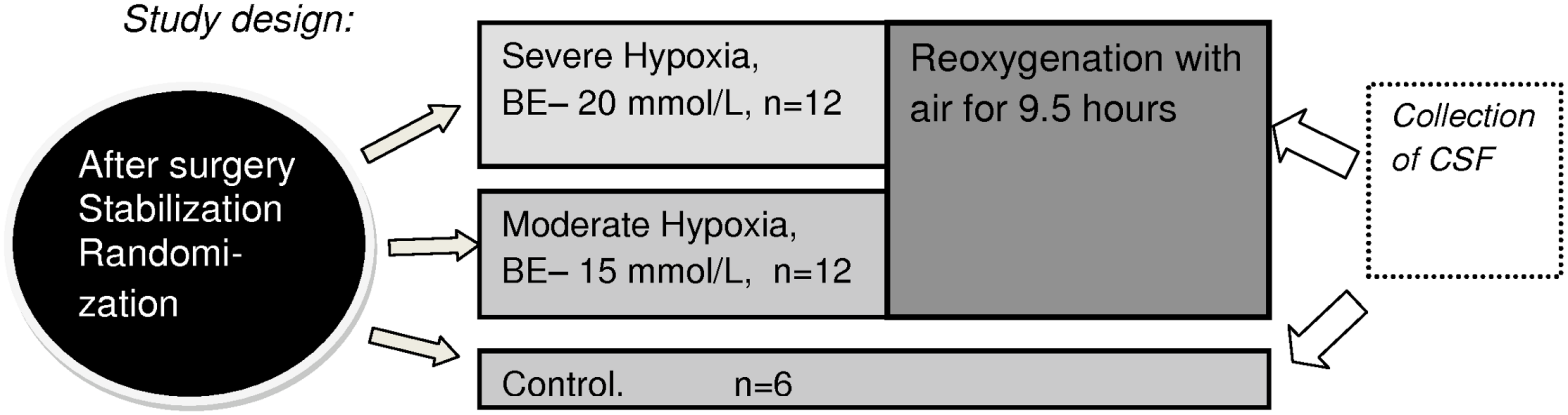
Study design. Twelve pigs were included in each experimental group and six pigs in the control group. The pigs in the first experimental group were exposed to global hypoxia (8% O2 in Nitrogen) until Base Excess (BE) reached -15 mmol/l (moderate hypoxia). The animals in the second experimental group (severe hypoxia) were exposed to 8% O2 until BE reached -20 mmol/l and/ or mean blood pressure fell below 20 mmHg. Thereafter the pigs were reoxygenated with air for 9.5 hours. CSF was collected 9.5 hours after end of hypoxia.

The pigs in the first experimental group were exposed to global hypoxia (8% O2 in Nitrogen) until Base Excess (BE) reached -15 mmol/l (moderate hypoxia). The animals in the second experimental group (severe hypoxia) were exposed to 8% O2 until BE reached -20 mmol/l and/ or mean blood pressure fell below 20 mmHg. During hypoxia, CO2 was added, aiming at a PaCO2 8.0–9.5 kPa, to imitate perinatal asphyxia. After the hypoxic challenge the pigs were reoxygenated with air and observed for 9.5 hours. The pigs in the control group were not exposed to hypoxia. Invasive blood pressure, EEG and ECG were measured continuously. The experiments were performed under Midazolam and Fentanyl anaesthesia, and all efforts were made to minimize suffering. Between 0.5 and 1.0 ml of CSF was collected via lumbar puncture with a 22G spinal needle from each pig at the end of the study and frozen at -70°C for further analysis (Fig 1). Blood for measuring Hb and S100B was collected before hypoxia and at the end of the study and frozen at -70°C. In addition arterial blood gases were collected at several time points throughout the experiment

### Method of sacrifice

After 9.5 hours of reoxygenation the pigs were subjected to euthanasia with an overdose of Pentobarbitone (150mg/kg).

### Approval

The Norwegian Council for Animal Research approved the experimental protocol (approval number 4630). The animals were cared for and handled in accordance with the European Guidelines for the use of experimental animals by researchers who have been certified by the Federation of European Laboratory Animals Science Association (FELASA).

### Sampling

CSF was sampled from 30 pigs via lumbar puncture. The quality of the CSF of three pigs was insufficient; therefore these were not included in the study. The samples were stored at -70°C until further analyzes. Then they were thawed, mixed and diluted and analyzed according to the manufacturers' instructions. For T-tau, p-Tau and AB42 ELISA kits were applied. (Innotest hTau Ag, Innotest Phospho-Tau (181P) and Innotest Beta-Amyloid; all Innogenetics, Gent, Belgium). S100B was measured using an electrochemiluminescent immunometric assay (ECLIA) on Cobas e 601 immunoassay platform (Roche Diagnostics,Mannheim,Germany). S100B cannot be assayed in EDTA-plasma, and thus serum was derived from the originally collected EDTA-plasma by adding 20 microL 2M CaCl_2_ to 1 mL sample. The samples were left to clot for 1 hour and centrifuged at 2500g for 15 minutes prior to assay of S100B. Neuron-specific Enolase was measured using the Kryptor NSE assay (Thermo Fischer Scientific B.R.A.H.M.S, Asnières, France), an automated homogenous immunometric assay based on the Time Resolved Amplified Cryptate Emission (TRACE) technology [11].

Brain tissue from hippocampus was homogenized in 5M guanidine HCl buffer with protease inhibitor and PMSF using Omnitip (Omni International USA). Homogenate was diluted with sample dilution buffer to 1:50 and 1:200. Final guanidine HCl concentrations were below 0.1 m. Sample duplicates were run on AB42 specific sandwich colorimetric ELISAs following the protocol of the manufacturer (BioSource, Camarillo, CA, USA). Optical densities at 450 nm of each well were read on a Multiscan Ascent (Thermo Scientific, MA, USA) and sample Aβ42 concentrations were determined by comparison with the AB42 standard curves.

### Immunohistochemistry

Four microm thick sections, which had been stored in 4% formaldehyde, were deparaffinized and rehydrated. The sections kept in citrate buffer, were heated in microwave for 15 min. The sections were then incubated with Formic acid 80% for 10 minutes, prior to blocking in a Peroxidase Block, 3% H2O2 for 10 minutes.

Following brief washes with Blocking buffer (5% goat serum, 5% BSA) for 30 minutes, the sections were incubated with Primary Antibody 6E10 (1:1000), a mouse monoclonal antibody, APP (Biolegend, MA, USA), and incubated over night at -4°C. The next day the slices were incubated with ImmPRESS (anti-mouse IgG, Vector Laboratories, CA, USA) for 30 minutes. For visualization DAB plus Peroxidase Substrate (Vector Laboratories, CA, USA) Kit were used.

### Expression analysis of Amyloid Protein Precursor (APP) from Hippocampus

Total RNA was extracted from tissue samples using the EZNA Total RNA Kit II (Omega Bio-Tek, Inc, Norcross GA, USA) according to manufacturers’ instructions.

Purified RNA was quantified using NanoDrop ND-1000 (NanoDrop Technologies, Delaware, USA) and 2 microg were reverse transcribed into cDNA with the High capacity cDNA Reverse Transcription kit (Applied Biosystems, Life Tech, CA, USA).

Primers were designed using Primer Express 3.0 Software (Applied Biosystems, USA).

Primer sequence: Forward: 5-´ CAGATCCGATCCCAGGTTATGA-3´.

Primer sequence: Reverse: 5-´AGCAGGAACGTTGTAGAGCAGG-3´.

A tenfold dilution of each primer showed efficiency of between 90% and 110%.

Amplification was performed for both target genes and reference gene P0 in a ViiA 7 Real Time PCR System, universal settings (Applied Biosystems, Life Tech, CA, USA).

An amount of 50 ng of each sample was run in duplicate with 400 nmol/l primers and Power SYBR Green Master Mix (Applied Biosystems, Warrington, UK).

Data were analyzed by the comparative Ct method (Delta-delta Ct method).

## Statistics

The data were statistically analyzed using the Kruskal-Wallis test and Mann-Whitney U test for variables with non-normal distributions, and independent samples t test when the distribution was normal. Levene´s test for equality of variance was performed before performing the t-test. If Levene´s test documented a significant variance difference between the compared groups, a t-test assuming different variances was performed. Otherwise, a t-test assuming equal variance was performed.

The biomarkers S100B and NSE were log-transformed to obtain a normal distribution.

Pearson´s correlation was used for calculation of the correlation between the log-values of NSE and S100B and between Lactate andAB42. The statistical analyses were performed by SPSS Statistics 19.0 (SPSS Inc., Chicago, IL, USA).

## Results

Comparing the two treatment groups of either moderate or severe hypoxia and the control group, there were no significant differences in weight, hemoglobin level, age, BE, PaCO2 or glucose level at start (Table 1). Table 2 describes the arterial blood gases, lactate and glucose taken at 5 different time points during the experiment. For one of the pigs in the group exposed to moderate hypoxia the BP fell below 20mmHg before reaching the predestined value of BE = -15mmol/l, (BE = -11mmol/l).

**Table 1:** Physiological parameters in the animals of the different groups at start.

GROUP	SEVERE HYPOXIA N = 12	MODERATE HYPOXIA N = 12	CONTROL N = 6
Weight (g)	1924 (124)	1982 (140)	1923 (76)
Haemoglobin g/dl	7.0 (1.0)	7.8(0.9)	7.7(1.8)
Gender (male/female)	6/6	6/6	3/3
Duration of Hypoxia (min)	33(12)	32 (9)	0
Age (h)	28.6 (3.5)	26.3 (4.6)	22.5 (1.5)
BE (mmol/l) Start	-0.3 (3.6)	1.6 (5.5)	4.3 (2.9)
Lactate (mmol/l)Start	2.8 (1.0)	1.8 (0.5)	2,3 (1,1)
Arterial pH Start	7.45 (0.04)	7,45 (0.07)	7,44 (0,12)
PaCO2 (kPa) Start	5.0 (1.1)	5.7 (1.4)	5.0 (1.1)
Glucose (mmol/l)	6.7 (2.3)	6.0 (0.9)	5,0 (1,2)

Between the different groups of the study cohort, there were no significant differences in weight, Hb level, age, BE, PaCO2 or glucose at start. Values are presented as mean (+/- SD) BE: base excess.

**Table 2:** BE, pH, PaCO2, Lactate and Glucose at 5 different time points after hypoxia.

BE, mmol/L	End hypoxia	30 min reox	90 min reox	270 min reox	570 min reox
Sev.Hypoxia	-19.0 (3.9)	-15.1 (3.5)	-5.7 (4.6)	-1.4 (5.0)	-3.7 (5.4)
Mod.Hypoxia	-15.8 (1.9)	-10.4 (2.8)	-1.3 (6.2)	-0.8 (5.7)	-4.0 (5.6)
Control	4.1 (3.6)	4.4 (1.9)	4.2 (2.3)	2.9 (4.5)	0.5 (3.4)
Arterial pH					
Sev. Hypoxia	6.92 (0.11)	7.18 (0.07)	7.35 (0.08)	7.39 (0.10)	7.39 (0.09)
Mod. Hypoxia	6.99 (0.05)	7.24 (0.06)	7.39 (0.10)	7.38 (0.06)	7.32 (0.10)
Control	7.43 (0.05)	7.46 (0.04)	7.45 (0.06)	7.42 (0.12)	7.38 (0.06)
PaCO2, kPa					
Sev.Hypoxia	7.7 (1.1)	4.4 (0.6)	4.6 (0.7)	5.2 (0.5)	4.5 (0.6)
Mod.Hypoxia	8.0 (1.7)	5.1 (0.7)	5.1 (0.8)	5.4 (0.5)	5.6 (1,3)
Control	5.7 (0.3)	5.4 (0.6)	5.5 (0.7)	5.5 (0.6)	5.9 (0.8)
Lactate, mmol/L					
Sev.Hypoxia	13.2 (3.1)	11.2 (0.6)	6.3 (2.1)	2.3 (2.2)	2.1 (2.0)
Mod.Hypoxia	11.9 (3.0)	9.4 (2.7)	3.9 (1.8)	1.7 (1.0)	2.3 (2.1)
Control	2.4 (2.1)	1.8 (1.1)	1.5 (0.6)	1.3 (0.4)	1.7 (1.0)
Glucose, mmol/L					
Sev.Hypoxia	9.1 (3.7)	7.5 (3.6)	6.7 (2.3)	4.9 (0.9)	4.7 (1.6)
Mod.Hypoxia	8.9 (3.4)	7.0 (3.1)	5.2 (1.6)	4.4 (1.2)	4.8 (1.4)
Control	5.0 (1.5)	4.7 (0.8)	4.9 (0.6)	4.3 (0.6)	4.3 (1.5)

Mean and (SD) for arterial blood gases (BE, pH, PaCO2, Lactate and Glucose) at end of hypoxia, 30, 90, 270 and 570 minutes after end of hypoxia. For the Control group the arterial blood gases stayed stable throughout the experiment. For the control group the described time points are corresponding time points.

At the end of hypoxia and 30 minutes after end of hypoxia there were significant differences between both intervention groups and the control group regarding the levels of BE, pH, lactate and glucose (p<0.05). Until 90 minutes after end of hypoxia there were still significant differences in all 4 parameters between the group exposed to severe hypoxia and the control group and BE and lactate remained significantly different until 120 minutes after end of hypoxia (p<0.05). Blood gases taken at later time points of recovery showed no significant differences between the groups.

### Cerebrospinal fluid

**Fig 2:**
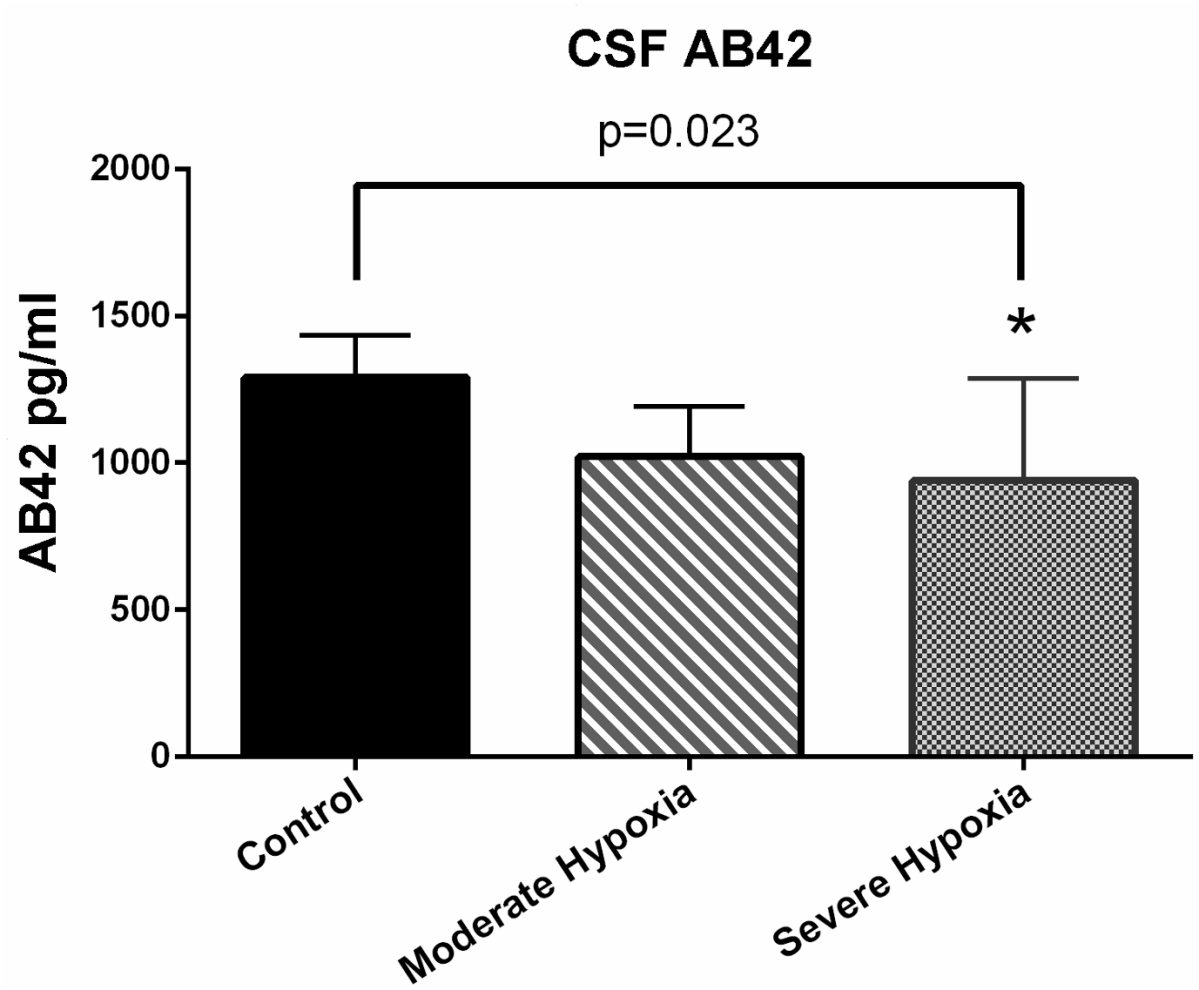
Mean values of the concentrations of AB42 with standard deviations (SD). AB42 was significantly lower in the group exposed to severe hypoxia * compared with the control group, 922 (SD +/-445) pg/ml vs. 1290 (SD +/-143) pg/ml, p<0.05. Mean difference was 368 (95% CI: 61–675). The concentration of AB42 in the group exposed to moderate asphyxia was 1059 (SD+/-75) pg/mg, p = 0.07, when compared with the control group.

Because of insufficient quality of CSF from 3 pigs, there were 5 pigs in the control group, 10 pigs in the group exposed to moderate hypoxia and 12 pigs in the group exposed to severe hypoxia. Fig 2 describes the level of AB42 in CSF for each group.

**Fig 3:**
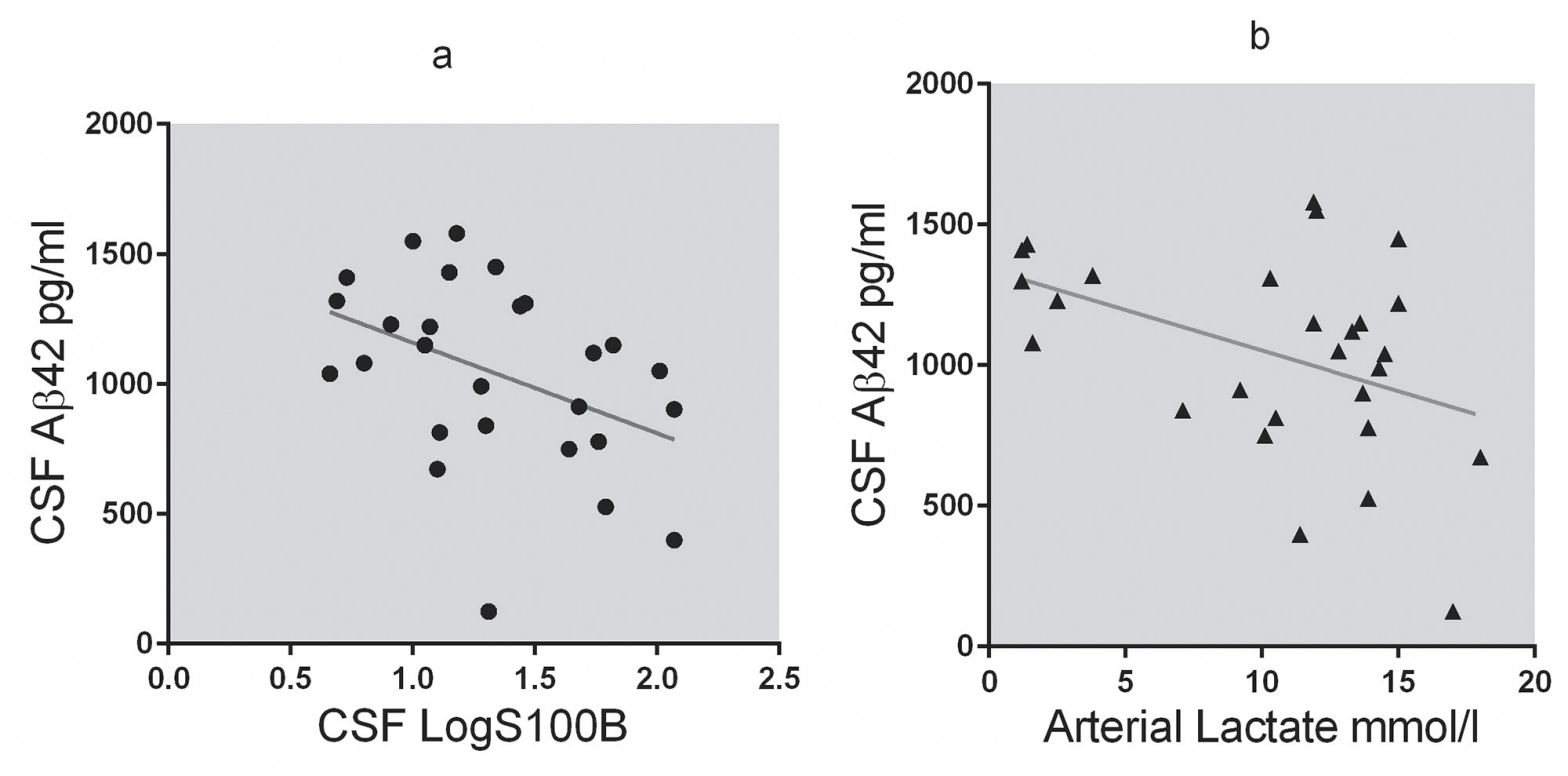
(A): Correlation between Log S100B and AB42 in CSF. There was a moderate negative correlation between LogS100B and AB42 in CSF, R = -0.418, p<0.05. (B): Correlation between Arterial Lactate and AB42 in CSF. Arterial Lactate at end of Hypoxia had a moderate negative correlation with AB42, R = -0.419, p = 0.03

There was a slight correlation between AB42 and LogS100B in CSF as well as AB42 in CSF and arterial Lactate (Fig 3A and 3B).

**Fig 4:**
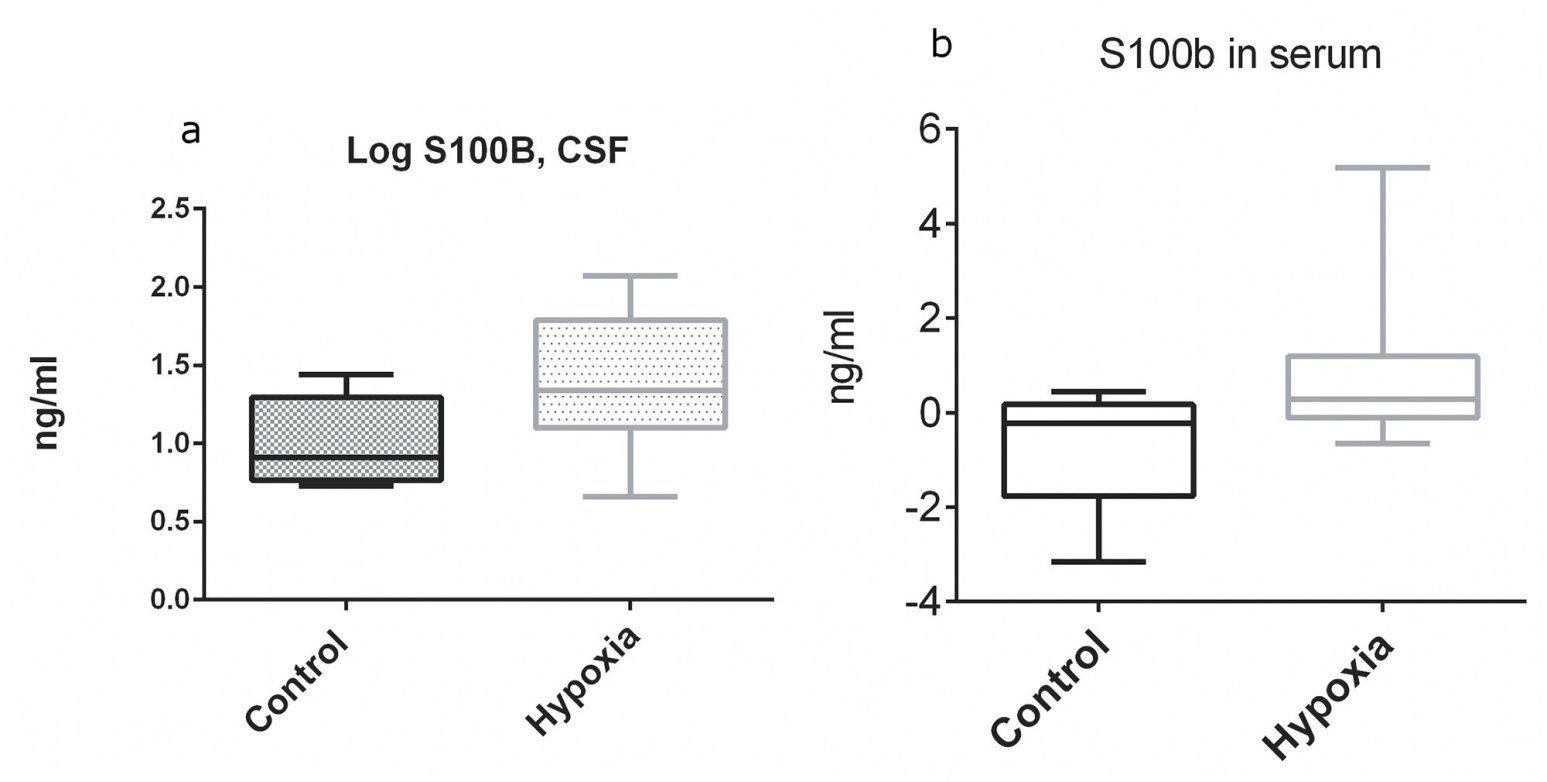
S100B in CSF and serum. (A) shows the Log-values of S100B in CSF for both intervention groups and the Control group, Log-S100B was significantly higher in the group exposed to hypoxia than in the control group, 1.0 (SD +/-0.3) pg/ml vs. 1.4 (SD +/-0.4) pg/ml, p<0.05. Mean difference was 0.4 (95% CI: 0.2–0.9). (B) depicts the Delta-Value from End of Hypoxia to End of experiment, p = 0.05. There was no difference between the group exposed to moderate vs. severe hypoxia regarding the levels of S100B, neither in CSF nor in blood, therefore we decided to combine both hypoxia groups into one intervention group.

We measured the levels of S100B in CSF and serum and the concentration of NSE in CSF (Fig 4A and 4B).

**Fig 5:**
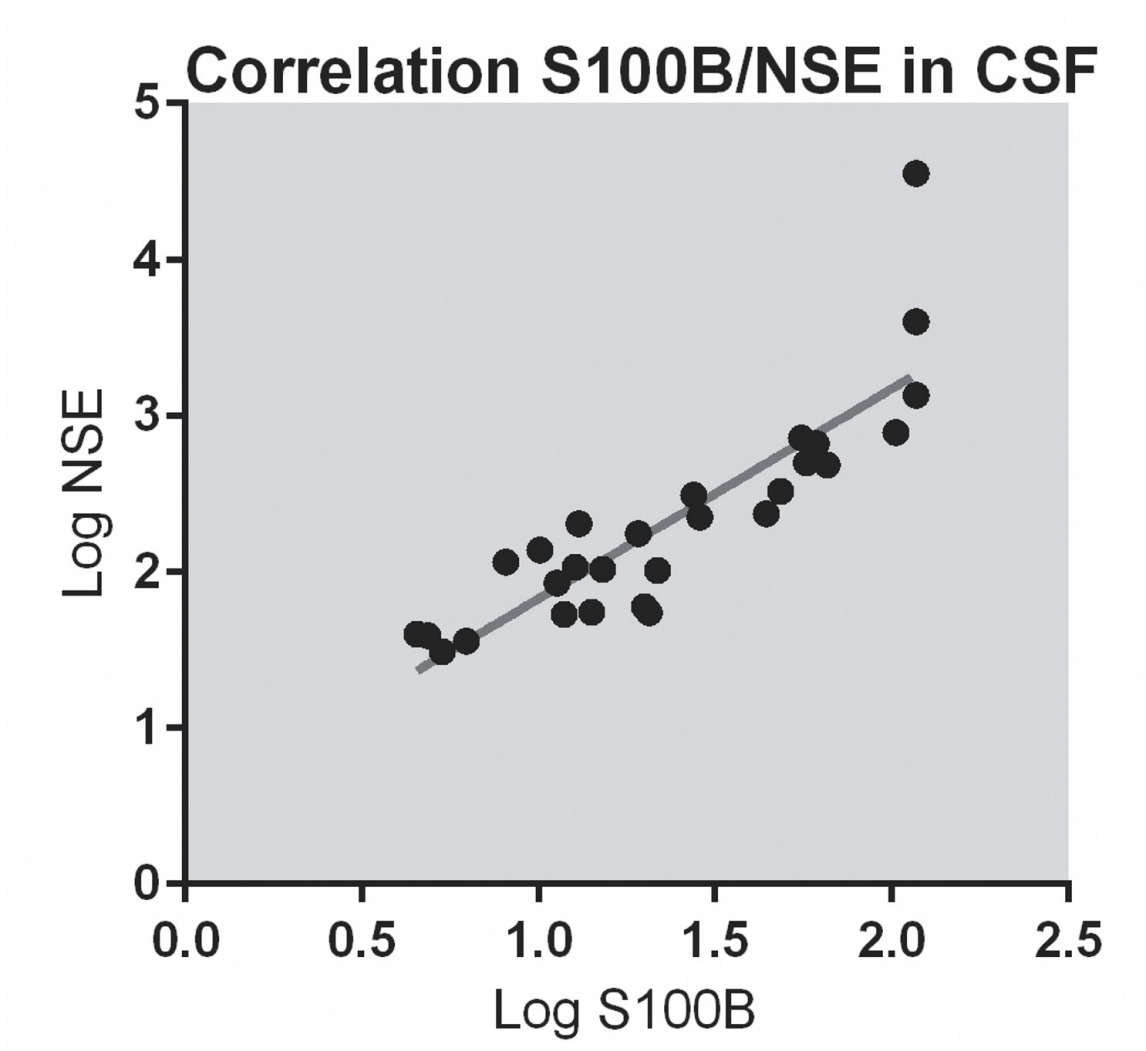
Correlation between NSE and S100B in CSF. Fig 5 describes the correlation between the logarithmic values of NSE and S100B in CSF. It was a high correlation between logNSE and logS100B measured in CSF at the end of the experiment, R = 0.86, p<0.001. The values are depicted as log-values because there was a relatively wide range in the concentration of NSE and S100B.

In spite of no significant difference of NSE in CSF between the hypoxia and control groups, p = 0.11, we found a strong correlation between the levels of NSE and S100B in CSF, (Fig 5).

(S1 and S2 Files are captions for supporting information files "correlations_LogNSE-Logs100b and Lactate-S100b.pzfx")

There were no differences between the intervention groups and the control group for T-tau and p-Tau in CSF.

### Brain tissue

AB42 could not be detected by ELISA in brain tissue homogenates and aggregated AB42 were not observed in any part of the brains by immunohistochemistry or in any of the slices and any of the animals examined.

The groups revealed no differences the gene expression of Amyloidal Precursor Protein in Hippocampus or Cortex.

## Discussion

The newborn pigs exposed to severe hypoxia revealed significantly lower levels of AB42 in CSF compared to the control group. To our knowledge the present study is the first to report a significant reduction in the level of AB42 in CSF and hypoxia-reoxygenation in a neonatal model.

A similar tendency was observed, although not significant, for those exposed to moderate hypoxia.

In accordance with the amyloid hypothesis decreased AB42 in CSF is supposed to be the first biomarker change to occur in AD [12,13]. A possible explanation for the absent T-tau and p-Tau changes in our experiment might be that the available limited time of 9.5 hours in our model is too short to develop tau changes. With longer observation time one might expect increased T-tau, an unspecific marker of neuronal injury.

AB42 and its protein precursor, Amyloid Beta protein precursor (B-APP) have played a central role in research on AD. However, the function of B-APP and AB in the nervous system is controversial.

B-APP-knockout mice show severe behavioral deficits, possibly indicating that B-APP has important physiological functions in the nervous system.

In the prodromal and preclinical stages of AD the level of AB42 in CSF is reduced [14] and we found a similar pattern in the present neonatal hypoxia-reoxygenation model. In AD, the level of soluble AB42 correlates with synaptic changes and disease severity [15], indicating an imbalance between production and clearance of AB42.

B, resulting in accumulation of toxic AB aggregates, neuroinflammation and neuronal cell death [16].

AB are known to self-assemble into oligomers, which are thought to be an important source of toxicity by damaging the neurons [17].

Lambert et al.(1998) presented in a mouse model that neurotoxins comprising oligomers of AB42 could kill neurons in hippocampus [18]. AB induces liposome fusion in vitro, which may suggest that AB in a non-fibrillar form may play a role in the progression of AD by directly disturbing the plasma membrane of neurons and altering its property [19]. Pillot. et al (1999) were able to show that in a primary culture from cortical neurons, AB could induce neurotoxicity via an apoptotic pathway [20].

In accordance to other studies we found that S100B in CSF was significantly higher for those exposed to hypoxia [21, 22]. However, a significant moderate negative correlation between AB42 and S100B in CSF was also observed.

We did not detect any signs of AB42 in the brain homogenate with ELISA, possibly due to a low concentration of AB42 in the brain of the newborn pigs.

Neither did we detect any signs of aggregation of AB42 in the brain tissue with the optical microscope, however a possible presence of small aggregates of AB42, below the detection limit of an optical microscope, cannot be excluded.

Taking these points into consideration, it is tempting to speculate that the reduction of AB42 in CSF and the negative correlation with S100B after neonatal hypoxia-reoxygenation, could be a sign of aggregation of AB42, which in turn attacks the neurons, triggering a long-lasting process.

In addition, it is an interesting observation that the same cognitive skills which are very often reduced for relatively well-functioning children after perinatal asphyxia are similar to the skills which are influenced in the earliest phases of AD, such as attention and visuospatial skills [23–25].

The moderate negative correlation between AB42 and lactate at end of hypoxia could strengthen the speculation that perinatal asphyxia might inflict neurodegenerative changes, as a study on newborn lambs from 2014 showed a high correlation between lactate 4 hours after asphyxia and histological degeneration of hippocampus 72 hours after asphyxia [26].

Could the neurons injured after neonatal hypoxia-reoxygenation be more prone to increased oxidative stress in late adulthood than their peers and could this make them more prone to neurodegenerative disorders such as AD?

It would be worthwhile to study whether AB42 and S100B in CSF could represent useful biomarkers at an early stage of brain damage after perinatal hypoxia-reoxygenation.

### Limitations of the study

We are aware that a relatively small number of animals were investigated. The pigs were sacrificed 9.5 hours after end of hypoxia, thus there were no long term follow-up.

It would have been highly interesting examining the brain of the pigs with electron microscope and search for possible changes in the neurons. However, to find the regions of interest, when we could not discover any changes in the optical microscope would have been very difficult.

As this is an animal study caution should be taken interpreting it on humans.

## Conclusion

To our knowledge, this study is the first to show an association between AB42 in CSF and perinatal hypoxia. Whether or not the reduction of AB42 in CSF after perinatal hypoxia has an etiological basis for adult neurodegenerative disorders needs to be studied with additional experiments and epidemiological studies.

AB42 and S100b are significantly changed in neonatal pigs subjected to severe hypoxia compared to controls, thus they may be valuable biomarkers of perinatal asphyxia.

## Supporting Information

S1 File Graphpad-File for the values of LogNSE and LogS100B for each pig. (PZFX)

S2 File Graphpad-File for the values of Lactate and S100B for each pig. (PZFX)
